# Role of copper and alumina for heat transfer in hybrid nanofluid by using Fourier sine transform

**DOI:** 10.1038/s41598-022-14936-x

**Published:** 2022-07-04

**Authors:** Basma Souayeh, Kashif Ali Abro, Ambreen Siyal, Najib Hdhiri, Faycal Hammami, Muayad Al-Shaeli, Nisrin Alnaim, S. Suresh Kumar Raju, Mir Waqas Alam, Tarfa Alsheddi

**Affiliations:** 1 Department of Physics, College of Science, King Faisal University, PO Box 400, Al Ahsa, 31982 Saudi Arabia; 2 Laboratory of Fluid Mechanics, Physics Department, Faculty of Science of Tunis, University of Tunis EI Manar, 2092 Tunis, Tunisia; 3 Department of Basic Sciences and Related Studies, Mehran University of Engineering and Technology, Jamshoro, Pakistan; 4 Institute of Ground Water Studies, Faculty of Natural and Agricultural Sciences, University of the Free State, Bloemfontein, South Africa; 5 Institute for Micro–Process Engineering (IMVT), Karlsruhe Institute of Technology, Hermann-von-Helmholtz-Platz 1, 76344 Eggenstein-Leopoldshafen, Germany; 6 Department of Mathematics and Statistics, College of Science, King Faisal University, P.O. Box 400, Al Ahsa, 31982 Saudi Arabia

**Keywords:** Fluid dynamics, Mechanical engineering

## Abstract

The convection, thermal conductivity, and heat transfer of hybrid nanofluid through nanoparticles has become integral part of several natural and industrial processes. In this manuscript, a new fractionalized model based on hybrid nanofluid is proposed and investigated by employing singular verses and non-singular kernels. The mathematical modeling of hybrid nanofluid is handled via modern fractional definitions of differentiations. The combined Laplace and Fourier Sine transforms have been configurated on the governing equations of hybrid nanofluid. The analytical expression of the governing temperature and velocity equations of hybrid nanofluid have been solved via special functions. For the sake of thermal performance, dimensional analysis of governing equations and suitable boundary conditions based on Mittage-Leffler function have been invoked for the first time in literature. The comparative analysis of heat transfer from hybrid nanofluid has been observed through Caputo-Fabrizio and Atangana-Baleanu differential operators. Finally, our results suggest that volume fraction has the decelerated and accelerated trends of temperature distribution and inclined and declined profile of heat transfer is observed copper and alumina nanoparticles.

## Introduction

Hybrid nanofluids showed enhanced characteristics of thermal conductivity in comparison with the conventional nanofluids; this is because hybrid nanofluids are a composite mixture of metallic, polymeric or non-metallic nano-sized power with base fluid used to improve the heat transfer rate in different applications. By Adding hybrid nanoparticles, one can significantly enhance thermal conductivity of the base fluid. It is worth noted that hybrid nanofluid can have higher heat transfer rate as compared to pure fluid as it is proved experimentally and numerically by numerous researchers^1,2^. It is established fact that metal nanoparticles exhibit unique properties that are highly appreciated in many novel-material applications, for instance, process of coatings, e.g., process of antibacterial, manufacturing of sensors and several others^1–6^. Suresh et al.^7^ analyzed the experimental aspects of heat transfer phenomena and friction factor in laminar flow of hybrid nanofluid with 0.1% volume fraction of Al_2_O_3_-Cu water. They conclude that Nusselt number as well as friction characteristic increases for considered hybrid nanofluid when compared to water and simple nanofluid containing the Al_2_O_3_ nanoparticles. Nine et al.^8^ investigated the thermal behavior of Al_2_O_3_-multiwall CNTs hybrid nanofluid dispersed in deionized water. They examined the thermal conductivity and dispersion quality for ground Multiwall CNTs and non-ground Multiwall CNTs. Moreover, greater thermal conductivity was observed for hybrid nanofluid compared to monotype nanofluid with same concentration of Al_2_O_3._ A numerical technique based on finite volume discretization method has been applied by Takabi and Shokouhmand^9^ to analyze the turbulent flow of Al_2_O_3_-Cu/water hybrid nanofluid, Al_2_O_3_/water nanofluid and pure water through a circular tube within their comparison. Sundar et al.^10^ studied the flow for turbulence with CNTs-Fe_3_O_4_/water hybrid nanofluid for enhancement of heat transfer. They emphasized high thermal conductivity and greater magnetic effects on the nano-composition of carbon nanotubes. Sheikholeslami and Shamlooei^11^ presented an analysis for thermal and magnetic behavior of Fe_3_O_4_-water nanofluid. They presented the impact of pertinent parameters and volume concentration. Here, the conclusion was deeply discussed for thermal radiations when buoyancy force is raised. A model of micropolar hybrid nanofluid under thermal and momentum slip condition has been investigated by Nadeem and Abbas^12^. They presented and compared different parameters on thermal and velocity profiles for micropolar hybrid nanofluid versus nanofluid. Sajid and Ali^13^ presented an excellent review on experimental and theoretical consideration of thermal conductivity, synthesizes of nanomaterials and stability for enhancement of hybrid nanofluids. Different study models were discussed for thermal conductivity of hybrid nanofluid and the improvement in rate of heat transfer. Fallah et al.^14^ investigated the governing equation for laminar flow of hybrid nanofluid over a porous rotating disc for the sake of numerical treatment. They present the variation in temperature distribution and velocity profile for different parameters by means of graphical illustration. Dinarvand et al.^15^ traced out semi-analytical solution of governing equation for mixed convection flow of hybrid nanofluid over a linearly stretching horizontal sheet near a stagnation point. Ali et al.^16^ investigated a fully developed flow of hybrid nanofluid based on permeable channel of two plates. Khashie et al.^17^ studied the magnetized flow of water-based hybrid nanofluid over a permeable disc. Their results for temperature, velocity, Nusselt number and skin friction were depicted for the affected suction as well as Joule heating. Besides these studies of hybrid nanofluid, there are few studies for hybrid nanofluid to enhance rate of heat transfer by using various technical studies embedded here with^18–26^. On the other hand, some recent attempts are hybrid based nanofluid flow over hydromagnetic slippery^27–29^, hybrid nanofluid flow over a slender stretching sheet with zero nanoparticles flux^18,30–32^ and few multi-dimensional approaches for nanofluid with different geometries^33–35^. For the sake of novelty, a new fractionalized model based on hybrid nanofluid is proposed and investigated by employing singular verses and non-singular kernels. The mathematical modeling of hybrid nanofluid is handled via modern fractional definitions of differentiations. The combined Laplace and Fourier Sine transforms have been configurated on the governing equations of hybrid nanofluid. The analytical expression of the governing temperature and velocity equations of hybrid nanofluid have been solved via special functions. For the sake of thermal performance, dimensional analysis of governing equations and suitable boundary conditions based on Mittage-Leffler function have been invoked for the first time in literature. The comparative analysis of heat transfer from hybrid nanofluid has been observed through Caputo-Fabrizio and Atangana-Baleanu differential operators. Finally, our results suggest that inclined and declined profile of heat transfer depends upon the use of copper and alumina nanoparticles.

## Fractionalized mathematical modeling of hybrid nanofluid

Unsteady, viscous and incompressible flow of hybrid nanofluid lying on a plate is considered, such hybrid nanofluid is suspended through an addition of copper and alumina nanoparticles in ethylene glycol. To deal with the flow of hybrid nanofluid, hybrid nanofluid is assumed over a plate which is at rest initially. When $t \to 0^{ + }$, the velocity is $u = u_{0} {\mathbf{E}}_{\alpha } \left( {at} \right)$. Here $u_{0}$ defines a free stream velocity of boundary. ${\mathbf{E}}_{\alpha } \left( {at} \right)$ represents Mittage-Leffler function and $t$ is time which cause the movement in terms of generalized exponential plate. The flow of hybrid nanofluid is induced and its velocity varies with time and distance from the plate. The governing equations for a hybrid nanofluid on the basis of assumptions are:

1 $$ \frac{\partial u}{{\partial x}} + \frac{\partial v}{{\partial y}} = 0,\frac{\partial u}{{\partial t}} = \frac{{\mu_{hnf} }}{{\rho_{hnf} }}\frac{{\partial^{2} u}}{{\partial y^{2} }} + \frac{{\left( {\rho \beta_{T} } \right)_{hnf} g\left( {T - T_{\infty } } \right)}}{{\rho_{hnf} }}, \frac{\partial T}{{\partial t}} = \frac{{{\mathcal{K}}_{hnf} }}{{\left( {\rho C_{p} } \right)_{hnf} }}\frac{{\partial^{2} T}}{{\partial y^{2} }} + \frac{{Q \left( {T - T_{0} } \right)}}{{\left( {\rho C_{p} } \right)_{hnf} }}, $$

Equation (1) is subjected to the imposed conditions as:

2 $$ \begin{array}{*{20}l} {y = 0: {\text{and }}t > 0, u = u_{0} {\mathbf{E}}_{\alpha } \left( {at} \right), T = T_{w} , } \hfill \\ {y \to \infty : {\text{and }}t > 0, u \to 0, T \to T_{\infty } .} \hfill \\ \end{array} $$

The surface and ambient temperature to the hybrid nanolufluid is $T_{w}$ and $T_{\infty }$ respectively. Here, $\left( {x,y} \right)$ is used to measure the distances and normal to surface, while $\left( {u,v} \right)$ defines the velocities in $x$ and $y$ directions. For the sake of simplicity, the thermo-physical parameters used in hybrid nanofluid are described in Appendix (A1–A8). The typical and rheological parameters defined in Appendix (A1–A8) are illustrated as, $\mu_{hnf}$, ${\mathcal{K}}_{hnf}$ and $\rho_{hnf}$ are the viscosity, thermal conductivity and density of the hybrid nanofluid respectively. $\mu_{f} , {\mathcal{K}}_{f}$ and $\rho_{f}$ are the base fluids. The heat capacity and thermal expansion coefficient are $C$ and $\beta$. While $Al_{2} O_{3}$ and $C_{u}$ are the nanoparticles and the subscripts $f,1$ and 2 used for base fluid. The symbolic form of the dynamic viscosity for base fluid is $\nu_{f}$. Dimensionless parameters of time, normal distance to the surface, temperature and velocity of the hybrid nanofluid are expressed in Eq. (3) as

3 $$\theta \left(y\right)=\frac{\widehat{T}-{T}_{\infty }}{{T}_{w}-{T}_{\infty }},t=a\widehat{t}, u=\frac{\widehat{u}}{{u}_{\infty }},y=\widehat{y}\sqrt{\frac{a}{{\nu }_{f}}},$$

Employing Eq. (3) into Eqs. (1–2), we arrive at

4 $$\frac{1}{{Q}_{0}}\left\{\frac{\partial u (y,t)}{\partial t}-{Q}_{1}Ri\theta (y,t)\right\}=\frac{{\partial }^{2}u \left(y,t\right)}{{\partial y}^{2}},$$

5 $$\frac{{P}_{r}}{{\mathcal{Q}}_{2}}\left\{\frac{\partial \theta \left(y,t\right)}{\partial t}-{\mathcal{Q}}_{3}\Delta \theta \left(y,t\right)\right\}=\frac{{\partial }^{2}\theta \left(y,t\right)}{{\partial y}^{2}}.$$

The imposed conditions are

6 $$\begin{array}{c}y=0: \mathrm{and }t>0, u={u}_{0}{\mathbf{E}}_{\alpha }\left(at\right), \theta =t, \\ y\to \infty : \mathrm{and }t>0, u\to 0, \theta \to 0. \end{array}$$

where, the rheological and functional letting parameters for Eqs. (4–5) are illustrated in Appendix (A9–A10). In Appendix (A9–A10), ${P}_{r}$ represents Prandtl number, $Ri$ shows mixed convection, $\Delta$ defines heat source, ${R}_{e}$ is Reynolds number and ${G}_{r}$ suggests Grashof number. In order to generalize the integer order Eqs. (4–5) for hybrid nanofluid into non-classical differentiations namely Caputo-Fabrizio differential operator^36^ and Atangana-Baleanu differential operator^37^, an energy and velocity Eqs. (4–5) can be transformed into fractionalized format with normalized functions as

7 $$\frac{{P}_{r}}{{\mathcal{Q}}_{2}}\left\{\frac{{\partial }^{\alpha }\theta \left(y,t\right)}{\partial {t}^{\alpha }}-{\mathcal{Q}}_{3}\Delta \theta \left(y,t\right)\right\}=\frac{{\partial }^{2}\theta \left(y,t\right)}{{\partial y}^{2}},$$

8 $$\frac{{P}_{r}}{{\mathcal{Q}}_{2}}\left\{\frac{{\partial }^{\beta }\theta \left(y,t\right)}{\partial {t}^{\beta }}-{\mathcal{Q}}_{3}\Delta \theta \left(y,t\right)\right\}=\frac{{\partial }^{2}\theta \left(y,t\right)}{{\partial y}^{2}},$$

9 $$\frac{1}{{Q}_{0}}\left(\frac{{\partial }^{\alpha }u\left(y,t\right)}{\partial {t}^{\alpha }}-{Q}_{1}Ri\theta \left(y,t\right)\right)=\frac{{\partial }^{2}u\left(y,t\right)}{{\partial y}^{2}},$$

10 $$\frac{1}{{Q}_{0}}\left(\frac{{\partial }^{\beta }u(y,t)}{\partial {t}^{\beta }}-{Q}_{1}Ri\theta (y,t)\right)=\frac{{\partial }^{2}u\left(y,t\right)}{{\partial y}^{2}}.$$

Here, $\frac{{\partial }^{\alpha }}{\partial {t}^{\alpha }}$ and $\frac{{\partial }^{\beta }}{\partial {t}^{\beta }}$ are non-classical differentiations namely Caputo-Fabrizio differential operator and Atangana-Baleanu differential operator have been defined in Appendix (A11–A12). The normalization functions for Appendix (A11–A12) are defined as $M\left(0\right)=M\left(1\right)=M\left(\alpha \right)=1$ and $M\left(0\right)=M\left(1\right)=M\left(\beta \right)=1$. The primary and foremost objective of non-classical differentiations is to assess a comprehensive analysis regarding the fractionalized heat transfer of flow of hybrid nanofluids with memory effects.

## Analytic solutions of fractionalized energy equation of hybrid nanofluid

Equations (7–8) are the fractionalized energy equation of hybrid nanofluid which subjected to the imposed conditions, mentioned in Eq. (6). To determine the fractionalized temperature field from Eqs. (7–8) with functional differential operators of CF and AB via integral transform, we employ Fourier sine transform on Eqs. (7–8) as:

11 $${T}_{s}\left(\xi ,t\right)\left({D}_{t}^{\alpha }+\frac{{\mathcal{Q}}_{2}}{{P}_{r}}{\xi }^{2}-{\mathcal{Q}}_{3}\right)=\frac{{\mathcal{Q}}_{2}}{{P}_{r}}\sqrt{\frac{2}{\pi }}\xi t$$

12 $${T}_{s}\left(\xi ,t\right)\left({D}_{t}^{\beta }+\frac{{\mathcal{Q}}_{2}}{{P}_{r}}{\xi }^{2}-{\mathcal{Q}}_{3}\right)=\frac{{\mathcal{Q}}_{2}}{{P}_{r}}\sqrt{\frac{2}{\pi }}\xi t,$$

For the sake of elimination of time variable involved in Eq. (11–12), the fractionalized temperature field is tackled through Laplace transform, the resulting expressions are:

13 $$\overline{{T}_{s}}\left(\xi ,s\right)=\frac{\xi {A}_{0}}{{s}^{2}} \sqrt{\frac{2}{\pi }} \frac{\left(s+{\mathcal{Q}}_{5}\right)}{\left(s{\mathcal{Q}}_{6}-{\mathcal{Q}}_{7}\right)},$$

14 $$\overline{{T}_{s}}\left(\xi ,s\right)=\frac{\xi {A}_{0}}{{s}^{2}} \sqrt{\frac{2}{\pi }} \frac{\left({s}^{\beta }+{\mathcal{Q}}_{9}\right)}{\left({s}^{\beta }{\mathcal{Q}}_{10}-{\mathcal{Q}}_{11}\right)},$$

Here, the letting parameters for Eqs. (13–14) are expressed as ${\mathcal{Q}}_{4}=\frac{1}{1-\alpha }$, ${\mathcal{Q}}_{5}={\alpha \mathcal{Q}}_{4},$ ${\mathcal{Q}}_{6}={P}_{r}{\mathcal{Q}}_{4}+{A}_{0}{\xi }^{2}+{A}_{1}{P}_{r}$, ${\mathcal{Q}}_{7}={A}_{1}{P}_{r}{\mathcal{Q}}_{5}+{A}_{0}{\xi }^{2}{\mathcal{Q}}_{5},$ and ${\mathcal{Q}}_{8}=\frac{1}{1-\beta }$, ${\mathcal{Q}}_{9}=\beta {\mathcal{Q}}_{8},$ ${\mathcal{Q}}_{10}={P}_{r}\beta +{A}_{0}{\xi }^{2}-{A}_{1}{P}_{r},$ ${\mathcal{Q}}_{11}={{A}_{1}P}_{r}{\mathcal{Q}}_{9}+{A}_{0}{\xi }^{2}{\mathcal{Q}}_{9}$. Inverting Eqs. (13–14) through Fourier sine transform and writing them in the equivalent form, we get

15 $$\overline{T}\left(y,s\right)=\frac{1}{{s}^{2}}-\frac{2}{\pi }\underset{0}{\overset{\infty }{\int }}\left(\frac{{\mathcal{Q}}_{6}-{A}_{0}{\xi }^{2}}{{\mathcal{Q}}_{6}}\right)\frac{\mathit{sin}\left(y\xi \right)}{\xi }\left(\frac{s+{\mathcal{Q}}_{12}}{{s}^{2}\left(s+{\mathcal{Q}}_{13}\right)}\right)d\xi ,$$

16 $$\overline{T}\left(y,s\right)=\frac{1}{{s}^{2}}-\frac{2}{\pi }\underset{0}{\overset{\infty }{\int }}\left(\frac{{\mathcal{Q}}_{9}-{A}_{0}{\xi }^{2}}{{\mathcal{Q}}_{9}}\right)\frac{\mathit{sin}\left(y\xi \right)}{\xi }\left(\frac{{s}^{\beta }+{\mathcal{Q}}_{14}}{{s}^{2}\left({s}^{\beta }+{\mathcal{Q}}_{15}\right)}\right)d\xi ,$$

Equation (15–16) have been functionalized for parametric letting variables as: ${\mathcal{Q}}_{12}=\frac{{\xi }^{2}{A}_{0}{\mathcal{Q}}_{5}-{\mathcal{Q}}_{7}}{{\mathcal{Q}}_{6}-{A}_{0}},{\mathcal{Q}}_{13}=-\frac{{\mathcal{Q}}_{7}}{{\mathcal{Q}}_{6}},{\mathcal{Q}}_{14}=\frac{{\xi }^{2}{A}_{0}{\mathcal{Q}}_{9}-{\mathcal{Q}}_{11}}{{\mathcal{Q}}_{10}-{\xi }^{2}{A}_{0}} \mathrm{and }{\mathcal{Q}}_{15}=-\frac{{\mathcal{Q}}_{11}}{{\mathcal{Q}}_{10}}$. Inverting Eqs. (15–16) through Laplace transform and expressing them into special functions, we investigate the final solutions of fractionalized energy equation of hybrid nanofluid as:

17 $$T\left(y,t\right)=t-\frac{4}{{\pi }^{2}\sqrt{{\mathcal{Q}}_{13}t}}\underset{0}{\overset{\infty }{\int }}\left(\frac{{\mathcal{Q}}_{6}-{A}_{0}{\xi }^{2}}{{\mathcal{Q}}_{6}}\right)\frac{\mathit{sin}\left(y\xi \right)}{\xi }\underset{0}{\overset{\sqrt{{\mathcal{Q}}_{13}t}}{\int }}{e}^{{\tau }^{2}-{\mathcal{Q}}_{13}\left(t-\tau \right)}d\xi +{\mathcal{Q}}_{13}\underset{0}{\overset{t}{\int }}t{e}^{{\mathcal{Q}}_{13}\left(t-\tau \right)}d\tau .$$

18 $$ \begin{aligned} T\left( {y,t} \right) & = t - \frac{2}{\pi }\mathop \smallint \limits_{0}^{\infty } \left( {\frac{{{\mathcal{Q}}_{10} - A_{0} \xi^{2} }}{{{\mathcal{Q}}_{10} }}} \right)\frac{{\sin \left( {y\xi } \right)}}{\xi }\mathop \smallint \limits_{0}^{t} \delta_{{{\mathcal{Q}}_{15} }} \left( { - {\mathcal{Q}}_{15} t^{\alpha } } \right)d\tau d\xi - \frac{2}{\pi }\mathop \smallint \limits_{0}^{\infty } \left( {\frac{{{\mathcal{Q}}_{10} - A_{0} \xi^{2} }}{{{\mathcal{Q}}_{10} }}} \right)\frac{{\sin \left( {y\xi } \right)}}{\xi } \\ & \quad \times \,\mathop \smallint \limits_{0}^{t} t^{\alpha - 1} {\varvec{\delta}}_{\alpha ,\alpha } \left( { - {\mathcal{Q}}_{15} t^{\alpha } } \right)\left( {t - \tau } \right)d\tau d\xi . \\ \end{aligned} $$

Equations (17–18) are the generalized non-integer order analytical solutions based on hybrid nanofluid for energy. Such equations can satisfy the imposed initial and boundary conditions, they can be verified by employing $t\to 0$ and $y\to \infty$.

## Analytic solutions of fractionalized velocity equation of hybrid nanofluid

Equations (9–10) are the fractionalized energy equation of hybrid nanofluid which subjected to the imposed conditions, mentioned in Eq. (6). To determine the fractionalized temperature field from Eqs. (9–10) with functional differential operators of CF and AB via integral transform, we employ Fourier sine transform on Eqs. (9–10) as:

19 $${u}_{s}\left(\xi ,t\right)\left({D}_{t}^{\alpha }+{\mathcal{Q}}_{0}{\xi }^{2}\right)-{\mathcal{Q}}_{1}{T}_{s}\left(\xi ,t\right)={\mathcal{Q}}_{0}\xi \sqrt{\frac{2}{\pi }} {u}_{0}{\mathbf{E}}_{\alpha }\left(at\right),$$

20 $${u}_{s}\left(\xi ,t\right)\left({D}_{t}^{\beta }+{\mathcal{Q}}_{0}{\xi }^{2}\right)-{\mathcal{Q}}_{1}{T}_{s}\left(\xi ,t\right)={\mathcal{Q}}_{0}\xi \sqrt{\frac{2}{\pi }} {u}_{0}{\mathbf{E}}_{\alpha }\left(at\right),$$

For the sake of elimination of time variable involved in Eq. (19–20), the fractionalized temperature field is tackled through Laplace transform, the resulting expressions are:

21 $$\overline{{u}_{s}}\left(\xi ,s\right)=\sqrt{\frac{2}{\pi }}\left(\frac{{u}_{0}{\mathcal{Q}}_{0}\xi \left(s+{\mathcal{Q}}_{1}\right){s}^{\alpha }}{s\left(s{\mathcal{Q}}_{6}+{\mathcal{Q}}_{0}{\xi }^{2}{\mathcal{Q}}_{1}\right)\left({s}^{\alpha }-a\right)}\right)+\left(\frac{{\mathcal{Q}}_{1}\left(s+{\mathcal{Q}}_{1}\right)}{s{\mathcal{Q}}_{6}+{\mathcal{Q}}_{0}{\xi }^{2}{\mathcal{Q}}_{1}}\right)\overline{{T}_{s}}\left(\xi ,s\right),$$

22 $$\overline{{u}_{s}}\left(\xi ,s\right)=\sqrt{\frac{2}{\pi }}\left(\frac{{u}_{0}{\mathcal{Q}}_{0}\xi \left({s}^{\alpha }+{\mathcal{Q}}_{10}\right){s}^{\alpha }}{s\left({s}^{\alpha }{\mathcal{Q}}_{15}+{\mathcal{Q}}_{0}{\xi }^{2}{\mathcal{Q}}_{10}\right)\left({s}^{\alpha }-a\right)}\right)+\left(\frac{{\mathcal{Q}}_{1}\left({s}^{\alpha }+{\mathcal{Q}}_{10}\right)}{{s}^{\alpha }{\mathcal{Q}}_{15}+{\mathcal{Q}}_{0}{\xi }^{2}{\mathcal{Q}}_{10}}\right)\overline{{T}_{s}}\left(\xi ,s\right),$$

Here, the letting parameters for Eqs. (13–14) are expressed as ${\mathcal{Q}}_{16}=\frac{2{\mathcal{Q}}_{0}{\mathcal{Q}}_{10}{\xi }^{2}}{{\mathcal{Q}}_{15}+{\mathcal{Q}}_{0}{\xi }^{2}} \mathrm{and} {\mathcal{Q}}_{17}=\frac{{\mathcal{Q}}_{0}{\xi }^{2}{\mathcal{Q}}_{10}}{{\mathcal{Q}}_{15}}$. Inverting Eqs. (21–22) through Fourier sine transform and writing them in the equivalent form, we get

23 $$ \begin{aligned} \overline{u}\left( {y,s} \right) & = \frac{{u_{0} s^{\alpha } }}{{s\left( {s^{\alpha } - a} \right)}} - \frac{{2u_{0} }}{\pi }\mathop \smallint \limits_{0}^{\infty } \left( {\frac{{{\mathcal{Q}}_{6} + {\mathcal{Q}}_{0} \xi^{2} }}{{{\mathcal{Q}}_{6} }}} \right)\frac{{\sin \left( {y\xi } \right)}}{\xi }\frac{{s^{\alpha } \left( {s + {\mathcal{Q}}_{7} } \right)}}{{s\left( {s^{\alpha } - a} \right)\left( {s + {\mathcal{Q}}_{8} } \right)}}d\xi \\ & \quad + \,\frac{2}{\pi }\mathop \smallint \limits_{0}^{\infty } \left( {\frac{{{\mathcal{Q}}_{2} {\mathcal{Q}}_{1} \xi }}{{{\mathcal{Q}}_{6} {\mathcal{Q}}_{2} }}} \right)\frac{{sin\left( {y\xi } \right)}}{\xi } \times \frac{{\left( {s + {\mathcal{Q}}_{1} } \right)^{2} }}{{s^{2} \left( {s + {\mathcal{Q}}_{9} } \right)\left( {s - {\mathcal{Q}}_{5} } \right)}}d\xi , \\ \end{aligned} $$

24 $$ \begin{aligned} \overline{u}\left( {y,s} \right) & = \frac{{u_{0} s^{\alpha } }}{{s\left( {s^{\alpha } - a} \right)}} - \frac{{2u_{0} }}{\pi }\mathop \smallint \limits_{0}^{\infty } \left( {\frac{{{\mathcal{Q}}_{15} + {\mathcal{Q}}_{0} \xi^{2} }}{{{\mathcal{Q}}_{15} }}} \right)\frac{{\sin \left( {y\xi } \right)}}{\xi }\frac{{\left( {s^{\alpha } + {\mathcal{Q}}_{16} } \right) d\xi }}{{s\left( {s^{\alpha } - a} \right)\left( {s^{\alpha } + {\mathcal{Q}}_{17} } \right)}} \\ & \quad + \,\frac{2}{\pi }\mathop \smallint \limits_{0}^{\infty } \left( {\frac{{{\mathcal{Q}}_{2} {\mathcal{Q}}_{1} \xi }}{{{\mathcal{Q}}_{11} {\mathcal{Q}}_{15} }}} \right)\frac{{\sin \left( {y\xi } \right)}}{\xi } \times \frac{{\left( {s^{\alpha } + {\mathcal{Q}}_{10} } \right)^{2} d\xi }}{{s^{2} \left( {s^{\alpha } + {\mathcal{Q}}_{14} } \right)\left( {s^{\alpha } + {\mathcal{Q}}_{17} } \right)}}, \\ \end{aligned} $$

Inverting Eqs. (23–24) through Laplace transform and expressing them into special functions, we investigate the final solutions of fractionalized energy equation of hybrid nanofluid as via invoked Appendix (A13–A17):

25 $$ \begin{aligned} u\left( {y,t} \right) & = u_{0} {\varvec{E}}_{\alpha } \left( {at} \right) + \frac{{2u_{0} }}{\pi }\mathop \smallint \limits_{0}^{\infty } \frac{{\sin \left( {y\xi } \right)}}{\xi }\left( {\frac{{{\mathcal{Q}}_{6} + {\mathcal{Q}}_{0} \xi^{2} }}{{{\mathcal{Q}}_{6} }}} \right)\mathop \smallint \limits_{0}^{t} \left\{ {t^{\alpha } {\varvec{\delta}}_{\alpha ,\alpha } \left( {at^{\alpha } } \right)\left( {t - \tau } \right)^{\alpha } {\varvec{\delta}}_{1,1 - \alpha } \left( { - {\mathcal{Q}}_{8} \left( {t - \tau } \right)} \right)} \right\}d\tau d\xi \\ & \quad + \frac{{2u_{0} }}{\pi }\mathop \smallint \limits_{0}^{\infty } \frac{{\sin \left( {y\xi } \right)}}{\xi }\left( {\frac{{{\mathcal{Q}}_{6} + {\mathcal{Q}}_{0} \xi^{2} }}{{{\mathcal{Q}}_{6} }}} \right)\mathop \smallint \limits_{0}^{t} \left\{ {e^{{ - {\mathcal{Q}}_{8} t}} {\varvec{\delta}}_{\alpha } \left( {a\left( {t - r} \right)^{\alpha } } \right)} \right\}drd\xi - \frac{2}{\pi }\mathop \smallint \limits_{0}^{\infty } \frac{{\sin \left( {y\xi } \right)}}{\xi }\left( {\frac{{{\mathcal{Q}}_{1} {\mathcal{Q}}_{0} {\mathcal{Q}}_{1} \xi }}{{{\mathcal{Q}}_{9} {\mathcal{Q}}_{6} {\mathcal{Q}}_{5} {\mathcal{Q}}_{2} }}} \right)d\xi \\ & \quad + \frac{2}{\pi }\mathop \smallint \limits_{0}^{\infty } \frac{{\sin \left( {y\xi } \right)}}{\xi }\left( {\frac{{{\mathcal{Q}}_{2} {\mathcal{Q}}_{1} \xi }}{{{\mathcal{Q}}_{6} {\mathcal{Q}}_{2} }}} \right)\left( {\frac{{2{\mathcal{Q}}_{1} {\mathcal{Q}}_{9} {\mathcal{Q}}_{5} - {\mathcal{Q}}_{1} \left( {{\mathcal{Q}}_{9} - {\mathcal{Q}}_{5} } \right)}}{{{\mathcal{Q}}_{9}^{2} {\mathcal{Q}}_{5}^{2} }}} \right)d\xi - \frac{2}{\pi }\mathop \smallint \limits_{0}^{\infty } \frac{{\sin \left( {y\xi } \right)}}{\xi }\left( {\frac{{{\mathcal{Q}}_{2} {\mathcal{Q}}_{1} \xi }}{{{\mathcal{Q}}_{6} {\mathcal{Q}}_{2} }}} \right)e^{{ - {\mathcal{Q}}_{9} t}} \\ & \quad \times \left( {\frac{{{\mathcal{Q}}_{9}^{2} - 2{\mathcal{Q}}_{1} {\mathcal{Q}}_{9} + {\mathcal{Q}}_{1} }}{{{\mathcal{Q}}_{9}^{2} \left( { - {\mathcal{Q}}_{5} - {\mathcal{Q}}_{9} } \right)}}} \right)d\xi - \frac{2}{\pi }\mathop \smallint \limits_{0}^{\infty } \frac{{\sin \left( {y\xi } \right)}}{\xi }\left( {\frac{{{\mathcal{Q}}_{2} {\mathcal{Q}}_{1} \xi }}{{{\mathcal{Q}}_{6} {\mathcal{Q}}_{2} }}} \right)\left( {\frac{{{\mathcal{Q}}_{5}^{2} + 2{\mathcal{Q}}_{1} {\mathcal{Q}}_{5} + {\mathcal{Q}}_{1} }}{{{\mathcal{Q}}_{5}^{2} \left( {{\mathcal{Q}}_{9} + {\mathcal{Q}}_{5} } \right) }}} \right)e^{{{\mathcal{Q}}_{5} t}} d\xi , \\ \end{aligned} $$

26 $$ \begin{aligned} u\left( {y,t} \right) & = u_{0} {\varvec{E}}_{\alpha } \left( {at} \right) - \frac{{2u_{0} }}{\pi }\mathop \smallint \limits_{0}^{\infty } \frac{{\sin \left( {y\xi } \right)}}{\xi }\left( {\frac{{{\mathcal{Q}}_{15} + {\mathcal{Q}}_{0} \xi^{2} }}{{{\mathcal{Q}}_{15} }}} \right)\mathop \smallint \limits_{0}^{t} \left\{ {t^{\alpha - 1} {\varvec{\delta}}_{\alpha ,\alpha } \left( { - {\mathcal{Q}}_{17} t^{\alpha } } \right){\varvec{\delta}}_{\alpha } \left( {a\left( {t - \tau } \right)} \right)} \right\}d\tau d\xi \\ & \quad + \frac{{2u_{0} }}{\pi }\mathop \smallint \limits_{0}^{\infty } \frac{{\sin \left( {y\xi } \right)}}{\xi }\left( {\frac{{{\mathcal{Q}}_{16} }}{{{\mathcal{Q}}_{17} }}} \right)\left( {\frac{{{\mathcal{Q}}_{15} + {\mathcal{Q}}_{0} \xi^{2} }}{{{\mathcal{Q}}_{15} }}} \right)\mathop \smallint \limits_{0}^{t} \left\{ {1 - {\varvec{\delta}}_{\alpha } \left( { - {\mathcal{Q}}_{17} t^{\alpha } } \right)\left( {t - \tau } \right)^{\alpha - 1} {\varvec{\delta}}_{\alpha ,\alpha } \left( {a\left( {t - \tau } \right)^{\alpha } } \right)} \right\} d\tau d\xi \\ & \quad + \frac{2}{\pi }\mathop \smallint \limits_{0}^{\infty } \frac{{\sin \left( {y\xi } \right)}}{\xi }\left( {\frac{{{\mathcal{Q}}_{2} {\mathcal{Q}}_{1} \xi }}{{{\mathcal{Q}}_{11} {\mathcal{Q}}_{15} }}} \right)\mathop \sum \limits_{n = 0}^{\infty } \mathop \sum \limits_{m = 0}^{\infty } \frac{{\left( { - {\mathcal{Q}}_{14} } \right)^{n} }}{{\left( { - {\mathcal{Q}}_{17} } \right)^{ - m} }}\frac{{t^{1 + \alpha n + \alpha m} }}{{\Gamma \left( {2 + \alpha n + \alpha m} \right)}}d\xi + \frac{4}{\pi }\mathop \smallint \limits_{0}^{\infty } \frac{{\sin \left( {y\xi } \right)}}{\xi }\left( {\frac{{{\mathcal{Q}}_{10} {\mathcal{Q}}_{2} {\mathcal{Q}}_{1} \xi }}{{{\mathcal{Q}}_{11} {\mathcal{Q}}_{15} }}} \right) \\ & \quad \times \mathop \sum \limits_{n = 0}^{\infty } \mathop \sum \limits_{m = 0}^{\infty } \frac{{\left( { - {\mathcal{Q}}_{14} } \right)^{n} }}{{\left( { - {\mathcal{Q}}_{17} } \right)^{ - m} }}\frac{{s^{1 + \alpha + \alpha n + \alpha m} }}{{\Gamma \left( {2 + \alpha + \alpha n + \alpha m} \right)}}d\xi + \frac{2}{\pi }\mathop \smallint \limits_{0}^{\infty } \frac{{\sin \left( {y\xi } \right)}}{\xi }\left( {\frac{{{\mathcal{Q}}_{10}^{2} {\mathcal{Q}}_{2} {\mathcal{Q}}_{1} \xi }}{{{\mathcal{Q}}_{11} {\mathcal{Q}}_{15} }}} \right)\mathop \sum \limits_{n = 0}^{\infty } \mathop \sum \limits_{m = 0}^{\infty } \frac{{\left( { - {\mathcal{Q}}_{14} } \right)^{n} }}{{\left( { - {\mathcal{Q}}_{17} } \right)^{ - m} }} \\ & \quad \times \frac{{t^{1 + 2\alpha + \alpha n + \alpha m} }}{{\Gamma \left( {2 + 2\alpha + \alpha n + \alpha m} \right)}}d\xi . \\ \end{aligned} $$

Equations (25–26) are the generalized non-integer order analytical solutions based on hybrid nanofluid for velocity field. Such equations can satisfy the imposed initial and boundary conditions, they can be verified by employing $t\to 0$ and $y\to \infty$.

## Numerical results and discussion

This section is dedicatedly written for the significant role of nanoparticles (copper and alumina) and base fluid ethylene glycol. Ethylene glycol is often used in the industry for the processing of nanofluids because it is highly viscous and accessible. Here, hybrid nanofluid is modeled via modern fractional definitions of differentiations and the mathematical models of energy and momentum are simplified by Laplace and Fourier Sine transforms. The comparative analysis of heat transfer and velocity profile from hybrid nanofluid have been observed through Caputo-Fabrizio and Atangana-Baleanu differential operators. The results mentioned below for heat transfer and velocity profile have captured a comparative analysis between the dynamics of ethylene glycol conveying alumina-copper nanoparticles.

### Physical significance of volume fraction

**Figure 1:**
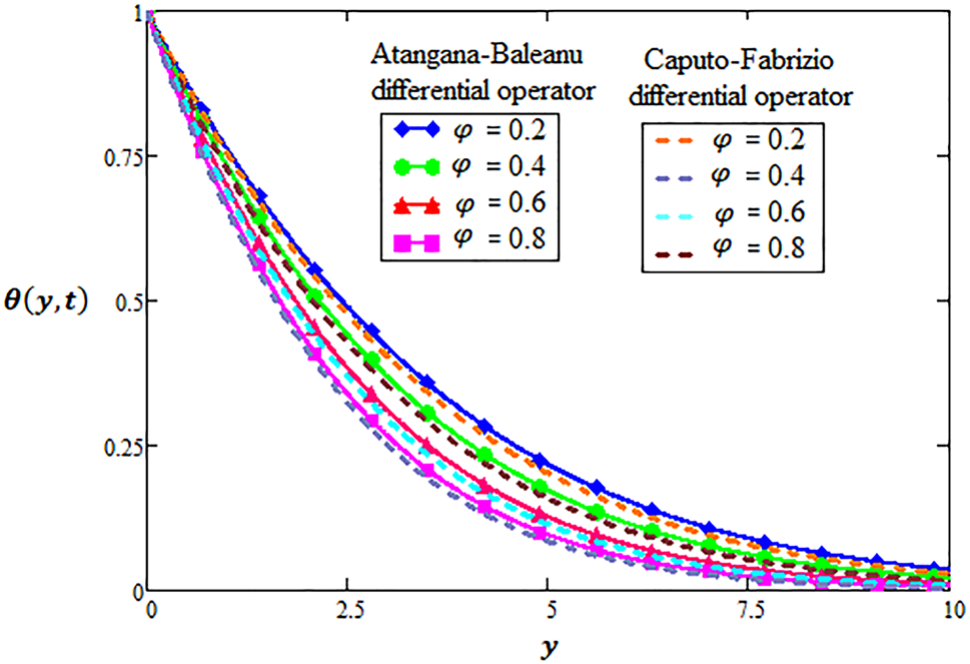
Variation of volume fraction for two-dimensional temperature distribution through AB-differential operator verses CF-differential operator.

**Figure 2:**
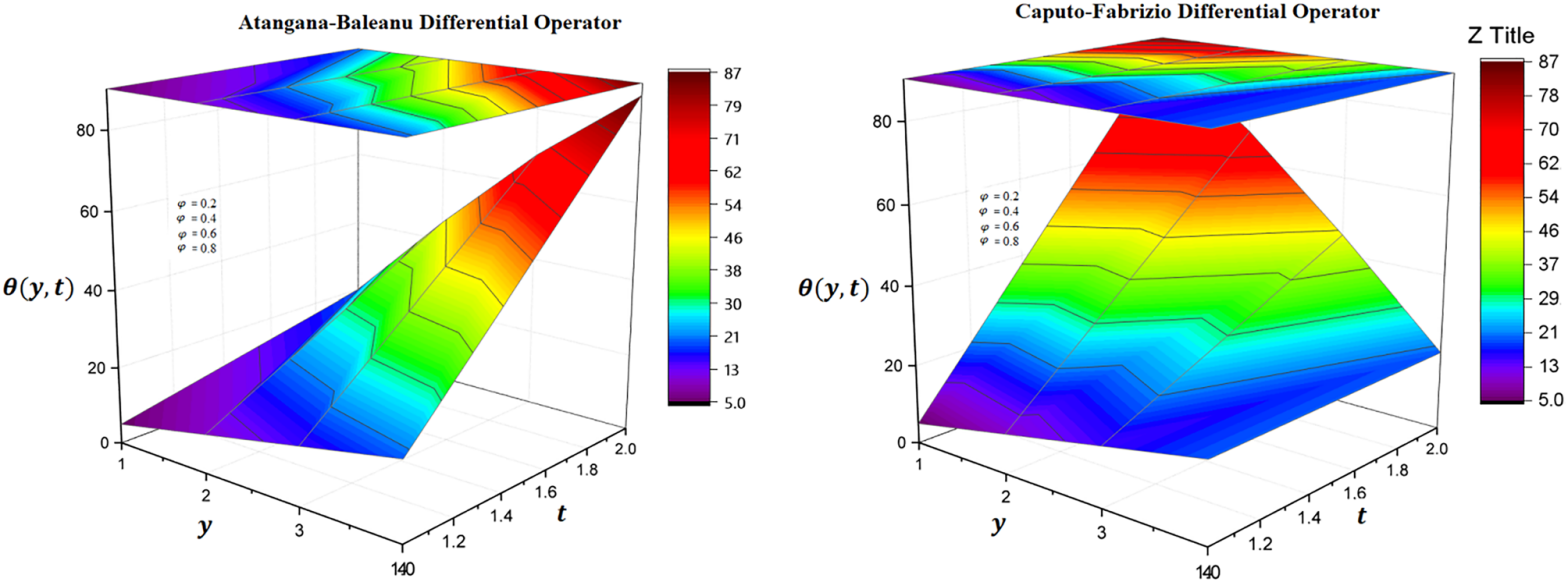
Variation of volume fraction for contour temperature distribution through AB-differential operator verses CF-differential operator.

**Figure 3:**
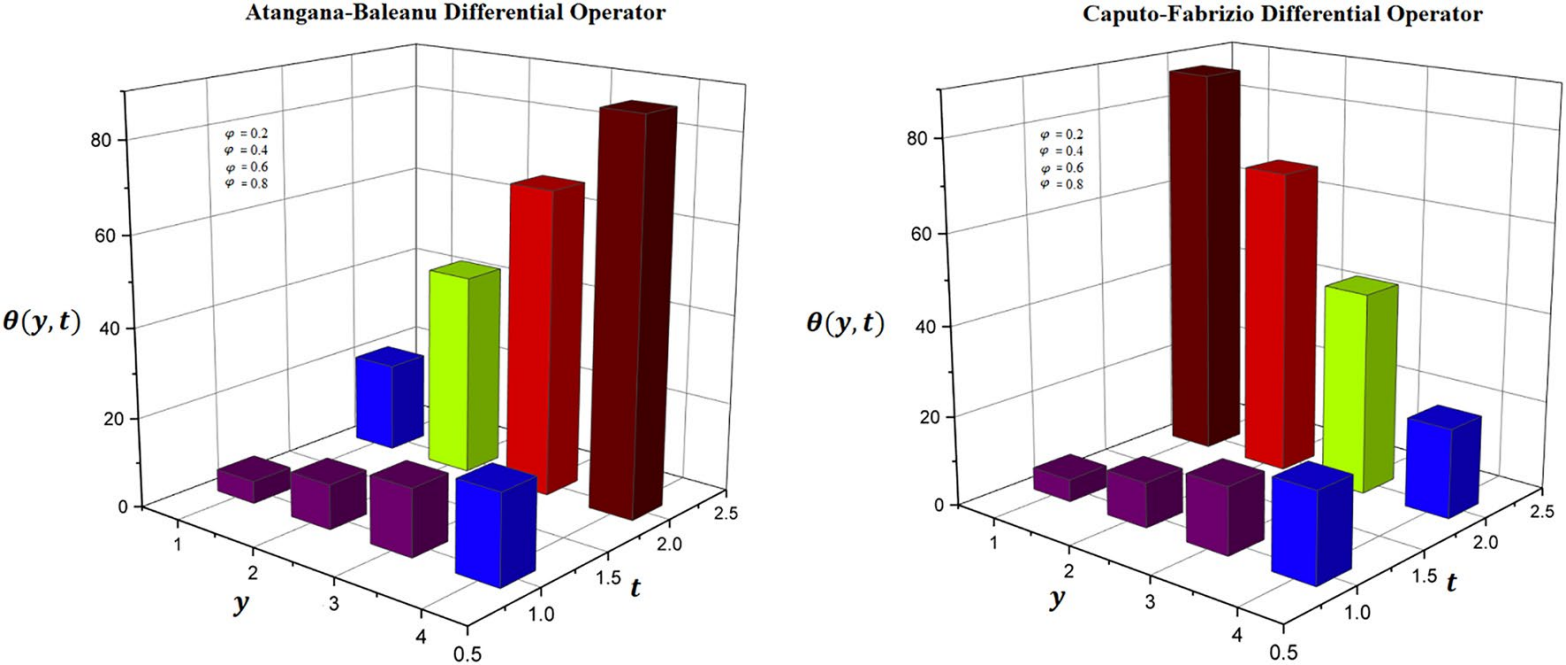
Variation of volume fraction for three-dimensional temperature distribution through AB-differential operator verses CF-differential operator.

It is established fact that when materials of similar dynamical characteristics are combined then volume fraction differences differ from the individual volumes of the mixture. In this context, temperature distribution has been investigated via modern fractional differential operators in Fig. 1 in which it is observed that increasing rate of volume fraction, the temperature distribution has decelerated trends through both differential techniques of fractional calculus. A selective range of volume fractions is sketched in Figs. 2 and 3 and numerically investigated for exceptional heat transfer to improve the effective thermal conductivity and thermal diffusivity of nanoparticle suspensions in base fluids. Additionally, Figs. 2 and 3 are prepared for contour plot and three-dimensional graphs for volume fraction of hybrid nanofluid respectively. It is observed that reciprocal behavior of temperature distribution is perceived for hybrid nanofluid.

### Physical significance of heat source parameter

**Figure 4:**
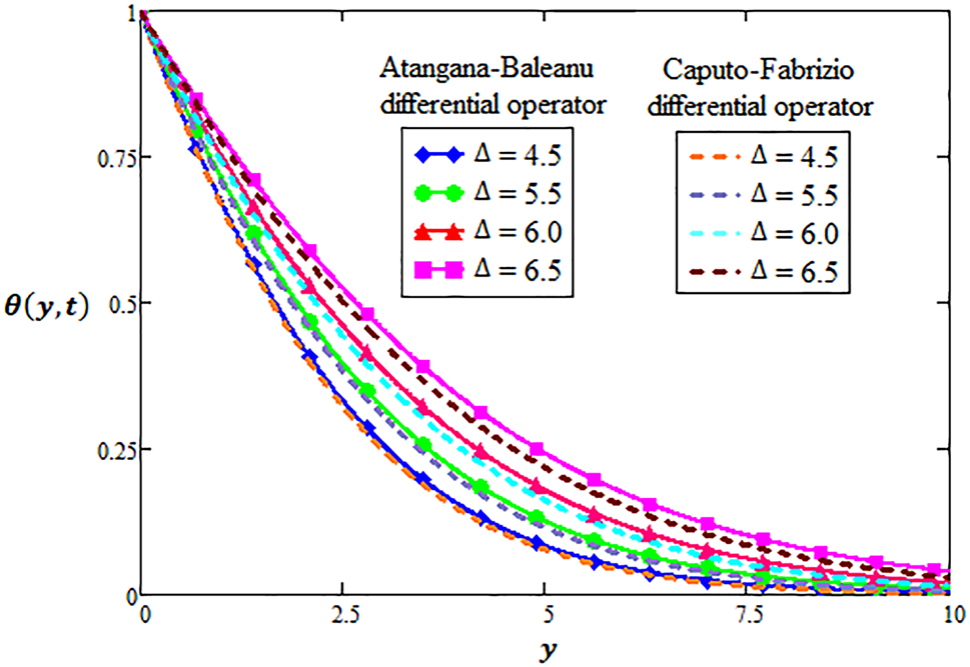
Variation of heat source for two-dimensional velocity profile through AB-differential operator verses CF-differential operator.

It is necessary to calculate the temperature distribution within the hybrid nanofluid when heat sources are present in the hybrid nanofluid. This is because, heat source is an important factor in the hybrid nanofluid for enhancing the reactivity of hybrid nanofluid. In order to signify the effects of heat source at $\Delta = 4.5,5.5, 6.0,6.5$, temperature distribution of hybrid nanofluid is depicted in Fig. 4 via two types of fractional differentiations. Here, it is perceived that enhancing the heat source raises heat transfer through both fractional techniques within sufficient uniformity. In short, from physical aspects, fractional techniques have envisaged the temperature distribution subject to the employment of heat source in symmetry.

### Physical significance of Grashof number

**Figure 5:**
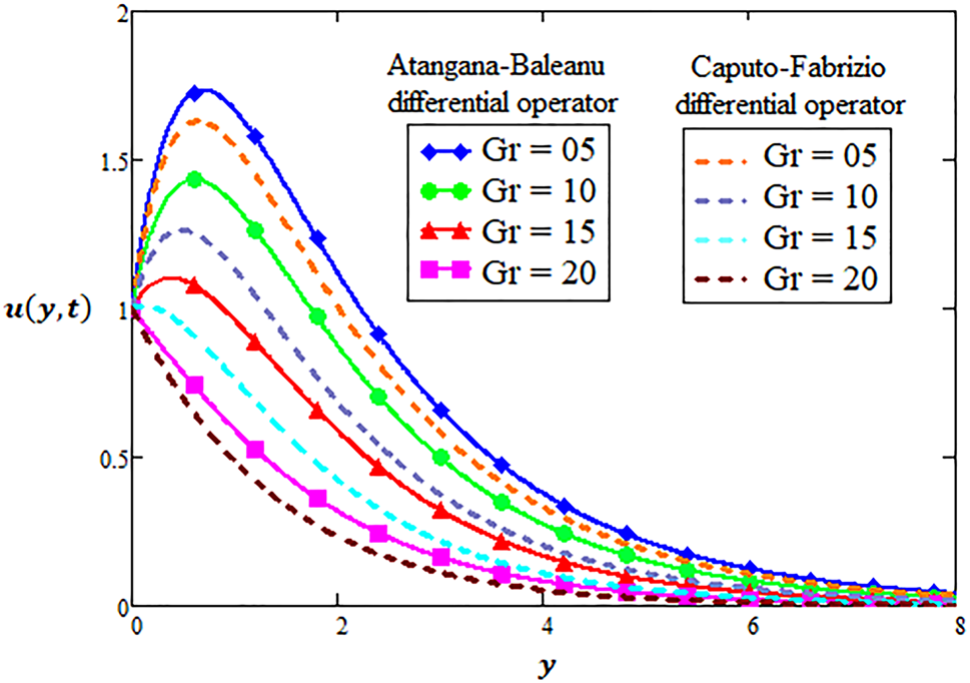
Variation of Grashof number for two-dimensional velocity profile through AB-differential operator verses CF-differential operator.

There is no denying fact that the Grashof number is a way to quantify the opposing forces. The profile of velocity is observed through both differential techniques of fractional calculus when Grashof number is varying at $Gr = 5,10,15,20$ in Fig. 5 for hybrid nanofluid. In is noticed from Fig. 5 that increasing Grashof number decreases velocity profile in free convection system. Physically, the larger values of Grashof number have reflected in which buoyant forces overcome the viscous forces. Hence, the flow starts a transition to the turbulent regime for hybrid nanofluid.

### Physical significance of Reynold number

**Figure 6:**
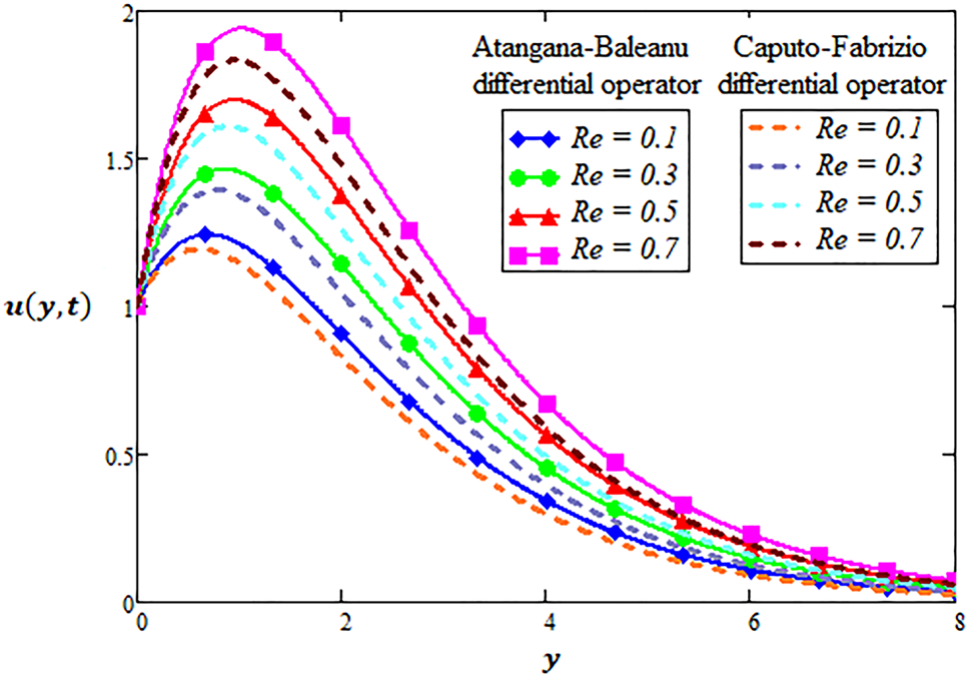
Variation of Reynold number for two-dimensional velocity profile through AB-differential operator verses CF-differential operator.

For flow from hybrid nanofluid over a flat plate, if Reynolds number is larger then inertia forces are in command. While viscous forces dominate the boundary layer when the Reynolds number is smaller. Keeping this fact for hybrid nanofluid, velocity field is sketched in Fig. 6 at the smaller values of Reynolds number via local and non-local kernel approach of differential operators. It is explored that velocity field raises when Reynolds number is increased. From practical point of view, the transport properties of hybrid nanofluid reacted to a variation in fluids from gases to liquids.

### Physical significance of suspension of nanoparticles in ethylene glycol

**Figure 7:**
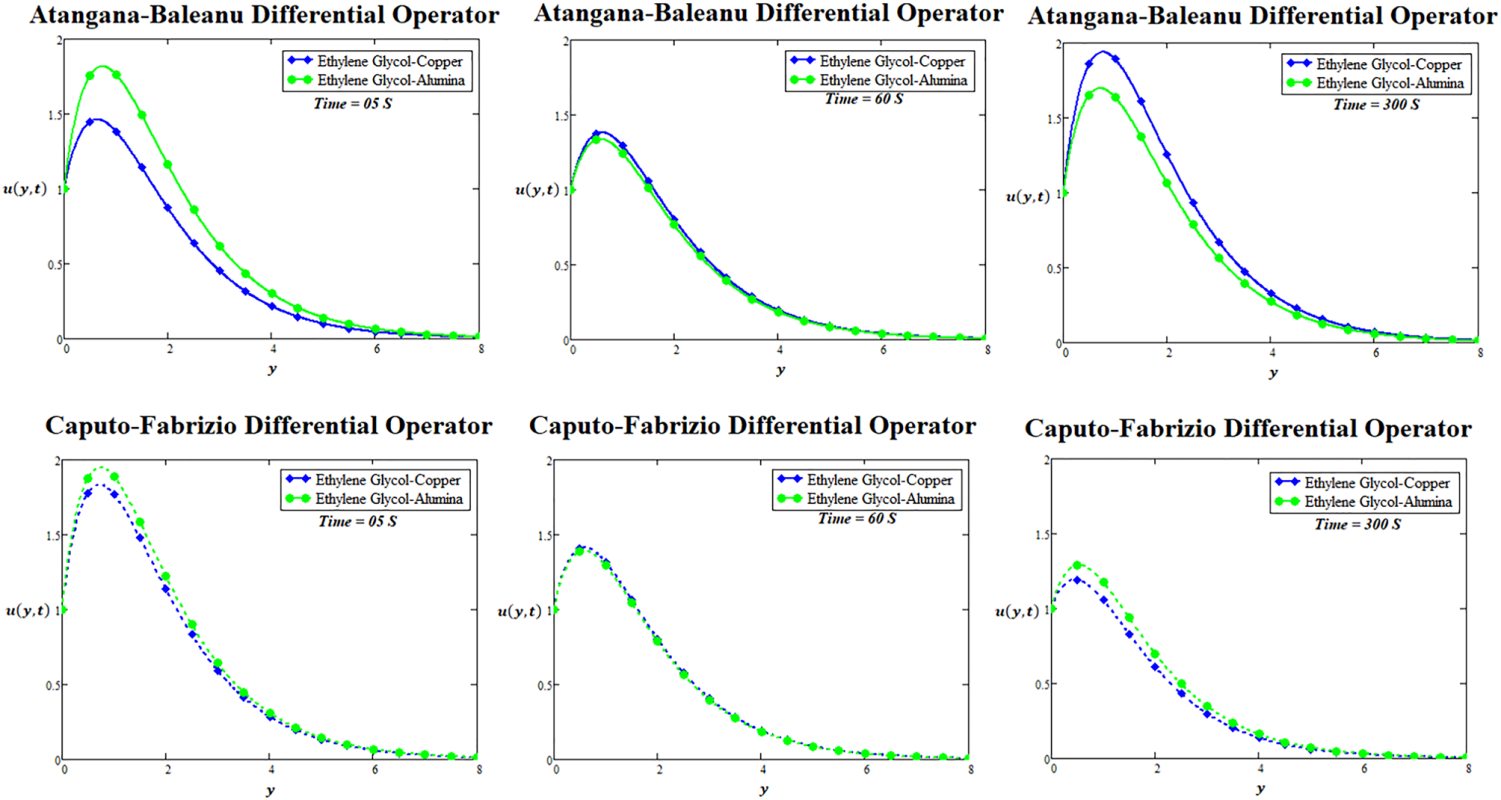
Comparison of copper and alumina nanoparticles suspended in ethylene-glycol for two-dimensional velocity profile through AB-differential operator verses CF-differential operator at three different times.

Due to extraordinary performance and high conductivity of copper and alumina nanoparticles in comparison with other nanoparticles, the copper and alumina nanoparticles are suspended in ethylene glycol. Copper nanoparticles may have similar principle of action as that of alumina nanoparticles; however, the precise mechanism regarding the suspension of both types of nanoparticles can cause viability and critical activity against the suspension of copper and alumina nanoparticles in ethylene glycol. Figure 7 is prepared for the profile of velocity for suspension of copper and alumina nanoparticles in ethylene glycol through Atangana-Baleanu and Caputo-Fabrizio fractional differential operators. Here, the comparative analysis of suspension of copper and alumina nanoparticles in ethylene glycol for velocity field is observed at three different times via both types of fractional differential operators. It is examined that ethylene glycol-alumina nanoparticles have faster velocity profile via both fractional differential operators at smaller time $t = 5 {\text{seconds}}$ in comparison with ethylene glycol-copper nanoparticles. While it is perceived that ethylene glycol-copper nanoparticles have faster velocity profile via Atangana-Baleanu fractional differential operator at lager time $t = 300 \,{\text{s}}$ in comparison with ethylene glycol-alumina nanoparticles. To conclude, identical trends of velocity profile is recognized for copper and alumina nanoparticles suspended in ethylene glycol at unit time $t = 60\, {\text{s}}$. For the sake of simplicity, the similar observation can also be investigated for temperature distribution.

## Conclusion

A new fractionalized model based on hybrid nanofluid is proposed and investigated by employing singular verses and non-singular kernels. The mathematical modeling of hybrid nanofluid is handled by invoking modern fractional definitions of differentiations. The combined Laplace and Fourier Sine transforms have been configurated on the governing equations of temperature and velocity of hybrid nanofluid. The analytical expression of the governing temperature and velocity equations of hybrid nanofluid have been solved via special functions. In brevity, following outcomes have been underlined as:

- Exact analytical solutions by invoking generalized non-integer order fractional differential operator have been investigated from basic equations of hybrid nanofluid. The investigated exact analytical solutions of temperature distribution [Eqs. (17–18)] and velocity field [Eqs. (25–26)] are stable for the proposed parametric values of fractional differential operators within the range $0 < \alpha ,\beta \le 1$.
- Increasing rate of volume fraction resulted the decelerated and accelerated trends of temperature distribution via Caputo-Fabrizio and Atangana-Baleanu fractional differential operators respectively.
- Temperature distribution of hybrid nanofluid is perceived by enhancing the heat source that raises heat transfer through both fractional techniques within sufficient uniformity.
- The larger values of Grashof number have reflected that buoyant forces overcome the viscous forces.
- Velocity field raises when Reynolds number is increased.
- The ethylene glycol-alumina nanoparticles have faster velocity profile via both fractional differential operators at smaller time $t = 5 \,{\text{s}}$ and ethylene glycol-copper nanoparticles have faster velocity profile via Atangana-Baleanu fractional differential operator at lager time $t = 300 \,{\text{s}}$.

## Data Availability

The datasets used and/or analysed during the current study available from the corresponding author on reasonable request.

## Appendix

A1 $${\mu }_{hnf}=\frac{{\mu }_{f}}{{\left(1-{\varphi }_{1}-{\varphi }_{2}\right)}^{2.5}},$$

A2 $${\rho }_{hnf}={\varphi }_{1}{\rho }_{1}+{\varphi }_{2}{\rho }_{2}+{\left(1-{\varphi }_{hnf}\right)}_{\rho f},$$

A3 $${\alpha }_{hnf}=\frac{{\mathcal{K}}_{hnf}}{{\left(\rho {C}_{p}\right)}_{hnf}},$$

A4 $${\varphi }_{hnf}={\varphi }_{1}+{\varphi }_{2},$$

A5 $${\left(\rho {C}_{p}\right)}_{hnf}={\varphi }_{1}{\left(\rho {C}_{p}\right)}_{1}+{\varphi }_{2}{\left(\rho {C}_{p}\right)}_{2}+\left(1-{\varphi }_{hnf}\right){\left(\rho {C}_{p}\right)}_{f},$$

A6 $$\frac{{\mathcal{K}}_{hnf}}{{\mathcal{K}}_{f}}=\frac{\left({\mathcal{K}}_{hp}+{2\mathcal{K}}_{f}\right)-{2\varphi }_{hnf}\left({\mathcal{K}}_{f}-{\mathcal{K}}_{hp}\right)}{\left({\mathcal{K}}_{hp}+{2\mathcal{K}}_{f}\right)+{\varphi }_{hnf}\left({\mathcal{K}}_{f}-{\mathcal{K}}_{hp}\right)},$$

A7 $${\mathcal{K}}_{hp}=\frac{{\varphi }_{1}{\mathcal{K}}_{1}+{{\varphi }_{2}\mathcal{K}}_{2}}{{\varphi }_{1}+{\varphi }_{2}},$$

A8 $${\left(\rho {\beta }_{T}\right)}_{hnf}={{\varphi }_{1}\left(\rho {\beta }_{T}\right)}_{1}+{{\varphi }_{2}\left(\rho {\beta }_{T}\right)}_{2}+\left(1-{\varphi }_{hnf}\right){\left(\rho {\beta }_{T}\right)}_{f},$$

A9 $$ {\mathcal{Q}}_{0} = \frac{{\frac{{\mu_{hnf} }}{{\mu_{f} }}}}{{\frac{{\rho_{hnf} }}{{\rho_{f} }}}}, {\mathcal{Q}}_{1} = \frac{{\left( {\rho \beta } \right)_{hnf} }}{{(\rho \beta )_{f} }} \frac{{\rho_{f} }}{{\rho_{hnf} }}, {\mathcal{Q}}_{2} = \frac{{\frac{{{\mathcal{K}}_{hnf} }}{{{\mathcal{K}}_{f} }}}}{{\frac{{\left( {\rho C_{p} } \right)_{hnf} }}{{\left( {\rho C_{p} } \right)_{f} }}}}, {\mathcal{Q}}_{3} = \frac{1}{{\frac{{\left( {\rho C_{p} } \right)_{hnf} }}{{\left( {\rho C_{p} } \right)_{f} }}}}, $$

A10 $${P}_{r}=\frac{{\nu }_{f}{\left(\rho C\right)}_{f}}{{\mathcal{K}}_{f}},Ri=\frac{{G}_{r}}{{Re}^{2}},\Delta =\frac{Q}{\left\{\omega {\left({\rho C}_{p}\right)}_{f}\right\}},{R}_{e}=\frac{{u}_{0}^{2}}{a{\upnu }_{f}},{G}_{r}=\frac{{\left(g{\beta }_{T}\right)}_{f}\left({T}_{w}-{T}_{\infty }\right){\left(\frac{{u}_{0}}{a}\right)}^{3}}{{\nu }_{f}^{2}},$$

A11 $$\frac{{\partial }^{\alpha }u\left(y,t\right)}{\partial {t}^{\alpha }}= \frac{M\left(\alpha \right)}{1-\alpha }\underset{0}{\overset{t}{\int }}\mathrm{exp}\left(\frac{-\alpha \left(z-t\right)}{1-\alpha }\right)u {}^{^{\prime}}\left(y,t\right)dt.$$

A12 $$\frac{{\partial }^{\beta }u\left(y,t\right)}{\partial {t}^{\beta }}= \frac{M\left(\beta \right)}{1-\beta }\underset{0}{\overset{t}{\int }}{E}_{\beta }\left(\frac{-\beta {\left(z-t\right)}^{\beta }}{1-\beta }\right)u {}^{^{\prime}}\left(y,t\right)dt.$$

A13 $${\mathcal{L}}^{-1}\left\{\frac{1}{{s}^{p}}\right\}=\frac{{t}^{p-1}}{\Gamma \left(p\right)}.$$

A14 $$\left(f*g\right)\left(t\right)=\underset{0}{\overset{t}{\int }}f\left(t\right)g\left(t-u\right)du.$$

A15 $${\mathcal{L}}^{-1}\left(\frac{{s}^{\alpha }}{s\left({s}^{\alpha }+a\right)}\right)={\mathbf{E}}_{\alpha }\left(-a{t}^{\alpha }\right).$$

A16 $${\mathcal{L}}^{-1}\left(\frac{{s}^{\alpha }}{\left(s-a\right)}\right)=-{t}^{\alpha }{\mathbf{E}}_{\mathrm{1,1}-\alpha }\left(\alpha t\right).$$

A17 $${\mathcal{L}}^{-1}\left(\frac{1}{\left({s}^{\alpha }+a\right)}\right)={t}^{\alpha -1}{\mathbf{E}}_{\alpha }\left(-a{t}^{\alpha }\right).$$

## References

[1] Choi SUS, Eastmoan JA (1995). Enhancing Thermal Conductivity of Fluids with Nanoparticles.

[2] Choi SUS, Zhang ZG, Yu W (2001). Anomalously thermal conductivity enhancement in nano-tube suspensions. J. Appl. Phys. Lett..

[3] Ai L, Vafai K (2005). An investigation of Stokes’ second problem for non-Newtonian fluids. Numer. Heat Transfer Part A.

[4] Pritchard D, McArdle CR, Wilson SK (2011). The Stokes boundary layer for a power-law fluid. J. Non-Newtonian Fluid Mech..

[5] Ilyas K, Kashif AA (2018). Thermal analysis in Stokes’ second problem of nanofluid: Applications in thermal engineering. Case Stud. Therm. Eng..

[6] Kashif AA, Mohammad MR, Ilyas K, Irfan AA, Asifa T (2018). Analysis of Stokes’ second problem for nanofluids using modern fractional derivatives. J. Nanofluids.

[7] Suresh S, Venkitaraj KP, Selvakumar P, Chandrasekar M (2012). Effect of Al2O3–Cu/water hybrid nanofluid in heat transfer. Exp. Thermal Fluid Sci..

[8] Nine MJ, Batmunkh M, Kim JH, Chung HS, Jeong HM (2012). Investigation of Al2O3-MWCNTs hybrid dispersion in water and their thermal characterization. J. Nanosci. Nanotechnol..

[9] Takabi B, Shokouhmand H (2015). Effects of Al_2_O_3_–Cu/water hybrid nanofluid on heat transfer and flow characteristics in turbulent regime. Int. J. Mod. Phys. C.

[10] Sundar LS, Sousa ACM, Singh MK (2015). Heat transfer enhancement of low volume concentration of carbon nanotube-Fe3O4/water hybrid nanofluids in a tube with twisted tape inserts under turbulent flow. J. Therm. Sci. Eng. Appl..

[11] Sheikholeslami M, Shamlooei M (2017). Fe_3_O_4_-H_2_O nanofluid natural convection in presence of thermal radiation. Int. J. Hydrogen Energy.

[12] Nadeem S, Abbas N (2018). On both MHD and slip effect in Micropolar Hybrid nanofluid past a circular cylinder under stagnation point region. Can. J. Phys..

[13] Sajid MU, Ali HM (2018). Thermal conductivity of hybrid nanofluids: a critical review. Int. J. Heat Mass Transf..

[14] Fallah B, Dinarvand S, Yazdi ME, Nademi Rostami M, Pop IMHD (2019). Flow and heat transfer of SiC-TiO2/DO hybrid nanofluid due to a permeable spinning disk by a novel algorithm. J. Appl. Comput. Mech..

[15] Dinarvand S, Rostami MN (2019). Improvement of drug delivery micro-circulatory system with a novel pattern of CuO-Cu/blood hybrid nanofluid flow towards a porous stretching sheet. Int. J. Numer. Methods Heat Fluid Flow.

[16] Ali A, Noreen A, Saleem S, Aljohani AF, Awais M (2020). Heat transfer analysis of Cu-Al_2_O_3_ hybrid nanofluid with heat flux and viscous dissipation. J. Therm. Anal. Calorim..

[17] Khashi'ie NS, Arifin NM, Nazar R, Hafidzuddin EH, Wahi N, Pop I (2020). Magnetohydrodynamics (MHD) axisymmetric flow and heat transfer of a hybrid nanofluid past a radially permeable stretching/shrinking sheet with Joule heating. Chin. J. Phys..

[18] Souayeh B, Ali Abro K, Alnaim N, Al Nuwairan M, Hdhiri N, Yasin E (2022). Heat transfer characteristics of fractionalized hydromagnetic fluid with chemical reaction in permeable media. Energies.

[19] Awan AU, Riaz S, Ashfaq M, Abro KA (2022). A scientific report of singular kernel on the rate-type fluid subject to the mixed convection flow. Soft Comput..

[20] Souayeh B, Abro KA, Alfannakh H, Al Nuwairan M, Yasin A (2022). Application of Fourier sine transform to carbon nanotubes suspended in ethylene glycol for the enhancement of heat transfer. Energies.

[21] Makinde OD, Khan WA, Khan ZH (2017). Stagnation point flow of MHD chemically reacting nanofluid over a stretching convective surface with slip and radiative heat. Proc. Inst. Mech. Eng. Part E J. Process Mech. Eng..

[22] Abro KA, Souayeh B, Malik K, Atangana A (2022). Chaotic characteristics of thermal convection at smaller verse larger Prandtl number through fractal and fractional differential operators from nanofluid. Int. J. Model. Simul..

[23] Abro KA, Atangana A, Gomez-Aguilar JF (2021). Ferromagnetic chaos in thermal convection of fluid through fractal–fractional differentiations. J. Thermal Anal. Calorim..

[24] Souayeh B, Abro KA (2021). Thermal characteristics of longitudinal fin with Fourier and non-Fourier heat transfer by Fourier sine transforms. Sci. Rep..

[25] Abro KA, Atangana A (2021). A computational technique for thermal analysis in coaxial cylinder of one-dimensional flow of fractional Oldroyd-B nanofluid. J. Ambient Energy Int..

[26] Abro KA, Gomez-Aguilar JF (2021). Fractional modeling of fin on non-Fourier heat conduction via modern fractional differential operators. Arab. J. Sci. Eng..

[27] Shib SG, Kalidas D, Prabir KK (2021). Computational analysis of thermal and mass convection in hybrid based nanofluid flow over hydromagnetic slippery curved surface. Int. J. Ambient Energy.

[28] Riaz S, Sattar M, Abro KA, Ali Q (2022). Thermo-dynamical investigation of constitutive equation for rate type fluid: a semi-analytical approach. Int. J. Model. Simul..

[29] Panhwer LA, Abro KA, Memon IQ (2022). Thermal deformity and thermolysis of magnetized and fractional Newtonian fluid with rheological investigation. Phys. Fluids.

[30] Kalidas D, Shib SG, Prabir KK (2021). Influence of Hall current effect on hybrid nanofluid flow over a slender stretching sheet with zero nanoparticles flux. Heat Transf..

[31] Abro KA, Atangana A, Memon IQ (2022). Comparative analysis of statistical and fractional approaches for thermal conductance through suspension of ethylene glycol nanofluid. Braz. J. Phys..

[32] Aysha R, Azad H, Sohail N (2021). Physical aspects of convective and radiative molecular theory of liquid originated nanofluid flow in the existence of variable properties. Phys. Scr..

[33] Azad H, Aysha R, Naqash A, Ahmed SE, Sahar AN, Abdulrazak HA, El-Sayed MS (2022). Heat transfer and flow characteristics of pseudoplastic nanomaterial liquid flowing over the slender cylinder with variable characteristics. Crystals.

[34] Ahmad S, Nadeem S, Rehman A (2021). Mathematical analysis of thermal energy distribution in a hybridized mixed convective flow. J. Nanofluids.

[35] Awan AU, Riaz S, Abro KA, Siddiqa A, Ali Q (2022). The role of relaxation and retardation phenomenon of Oldroyd-B fluid flow through Stehfest’s and Tzou’s algorithms. Nonlinear Eng. Model. Appl..

[36] Caputo M, Fabrizio M (2015). A new definition of fractional derivative without singular kernel. Progr. Fract. Differ. Appl..

[37] Atangana A, Baleanu D (2016). New fractional derivatives with nonlocal and nonsingular kernel: theory and application to heat transfer model. Therm. Sci..

